# Meningococcal carriage in adolescents and young adults in Kuwait

**DOI:** 10.3389/fpubh.2026.1766703

**Published:** 2026-04-24

**Authors:** Mohammed Alghounaim, Hamad Bastaki, Mohammed Egaila, Jennifer Onwumeh-Okwundu, Mohammed Abdel Aziz, Jean Joury, Lidia Sierra, Florence Lefebvre D’Hellencourt

**Affiliations:** 1 Department of Pediatrics, Amiri Hospital, Kuwait City, Kuwait; 2 Department of Public Health, Ministry of Health, Kuwait City, Kuwait; 3 Pfizer Gulf FZ LLC, Dubai, United Arab Emirates; 4 RWE Platform Pfizer Inc., Collegeville, PA, United States; 5 Pfizer Inc., New York, NY, United States

**Keywords:** Kuwait, meningococcal carriage, *Neisseria meningitidis*, public health, vaccination

## Abstract

**Background:**

*Neisseria meningitidis* is a major cause of invasive meningococcal disease (IMD), with asymptomatic carriage playing a crucial role in transmission. Adolescents (15–17 years) and young adults (18–20 years) have the highest carriage rates, yet data from Kuwait remain limited. This study aimed to estimate the prevalence of *N. meningitidis* carriage among adolescents and young adults in Kuwait.

**Methods:**

A cross-sectional study was conducted from July 2023 to May 2024, enrolling 1,398 participants aged 15–20 years. Randomly selected individuals provided oropharyngeal swabs, and demographic, behavioral, and vaccination data were collected via standardized questionnaires. Carriage prevalence was determined using selective culture-based methods. Whole genome sequencing was performed on culture-confirmed isolates. Descriptive statistics were used to summarize participant characteristics; carriage prevalence was reported as proportions, and associations were explored using Fisher’s exact or chi-square tests and logistic regression where feasible.

**Results:**

The overall meningococcal carriage rate was 0.21% (3/1,398). All isolates were non-groupable (NG), with one carrying the capsule null locus (CNL). Self-reported MenACWY vaccination coverage was 94.8%. No significant associations between demographic, geographic, or behavioral factors and carriage status were identified due to the small number of positive cases.

**Conclusion:**

This study provides a population-based estimate of meningococcal carriage among adolescents and young adults in Kuwait using a random sampling approach. The low carriage rate, alongside high vaccination coverage, suggests that existing immunization programs in Kuwait may be effective in reducing *N. meningitidis* transmission. However, the presence of NG strains stresses the need for continued surveillance. Further research is required to evaluate long-term vaccination impacts and potential gaps in disease protection.

## 1 Introduction

Invasive meningococcal disease (IMD), caused by *Neisseria meningitidis* (*N. meningitidis*), is a severe infection with a 5–15% mortality rate and long-term complications, including hearing loss and neurological damage (1, 2). Although *N. meningitidis* commonly colonizes the nasopharynx asymptomatically, it can invade the bloodstream or meninges, leading to septicemia or meningitis (1). The global burden remains significant; a 2013 review estimated an annual burden of approximately 1.2 million cases and 135,000 deaths worldwide (3). Among the 12 identified serogroups, six (A, B, C, W, X, Y) account for most invasive cases worldwide (1, 4, 5). In the Middle East, the distribution of meningococcal serogroups has varied by country and over time, with serogroups A, B, and W reported as predominant in different settings, including earlier reports from Iran, Kuwait, Qatar, Saudi Arabia, and Turkey (6).

Globally, adolescents and young adults (15–24 years) exhibit the highest carriage rates (10–35%), while other age groups show rates below 10% (7). Several behavioral and environmental factors, including smoking, alcohol use, intimate contact, and crowding (e.g., Hajj pilgrimage) contribute to carriage (8–12). This highlights the need for targeted vaccination and public health interventions (13, 14).

The duration of meningococcal carriage varies between individuals, with some maintaining the same strain, acquiring a replacement strain, or naturally achieving clearance (15–17). Although saliva-based testing is emerging, oropharyngeal swabbing remains the gold standard for carriage studies (18, 19). Most carried *N. meningitidis* strains are unencapsulated and non-pathogenic, as capsule expression is required for post-invasion survival (9, 20–24).

Kuwait has a low IMD incidence, with less than 10 cases per year (25, 26). However, the country remains at risk due to its geographic proximity to the meningitis belt, and high levels of migration. Despite inclusion of MenACWY vaccination in Kuwait’s national childhood immunization schedule (including a single dose at 12 months of age), challenges persist due to high population mobility and the influx of non-citizen workers who may not be covered by national programs in their home countries (27). While vaccination coverage is high among Kuwaiti nationals (28), international workers from low meningococcal risk countries may have lower immunization rates or may have received monovalent vaccines, which could contribute to potential gaps in disease protection (29). With 70% of its population comprising non-citizen workers, Kuwait faces additional hurdles in disease control (27).

In Kuwait, polyvalent (A, C, W, Y) conjugate meningococcal vaccine is routinely administered at 12 months and recommended for adults traveling to Mecca during Hajj. Furthermore, all children are screened for immunization status prior to school enrolment to ensure they are up to date with their vaccinations. Additionally, all individuals arriving from countries with high incidence of meningococcal disease (e.g., countries in the meningitis belt) are required to receive the vaccine prior to being granted residency. Similarly, children who have lived abroad and have not received the vaccine are also offered vaccination during the residency health check. The vaccine is also available at travel clinics for individuals traveling to areas at higher risk of transmission (30, 31).

Kuwait also maintains a robust surveillance system for meningococcal disease through timely detection, laboratory confirmation, and systematic reporting of cases. This comprehensive surveillance program plays a critical role in monitoring disease patterns, detecting outbreaks, and informing immunization strategies. However, data related to meningococcal carriage remains limited, highlighting the importance of conducting carriage studies to better understand transmission dynamics and guide public health interventions. Furthermore, disease epidemiology is poorly characterized in the country due to a paucity of epidemiological data. This study aims to estimate the prevalence of *N. meningitidis* carriage among adolescent and young adults in Kuwait, providing crucial insights into carriage dynamics in a highly vaccinated population.

## 2 Materials and methods

### 2.1 Study design and population

This cross-sectional study was conducted from July 2023 to May 2024, aiming to enroll 1,400 adolescents aged 15–20 years in Kuwait. Participants were recruited through a community household–based approach, rather than clinic-based sampling, using residential units registered in the national Public Authority of Civil Information (PACI) database. Residential units refer to registered household addresses within the national PACI sampling frame. Sampling was stratified by governorate and area of residence, based on the national registry of residential units maintained by the PACI, to ensure geographic representation across Kuwait. Each week, 2,500 residential units were selected using simple random sampling without replacement, and as many of these units as possible were approached for enrollment. Weekly sampling continued until 1,398 eligible participants were enrolled. Both Kuwaiti citizens and non-citizens were eligible for participation, irrespective of nationality.

Eligible participants were adolescents aged 15–20 years who resided in a selected unit, willing to provide an oropharyngeal sample, and had signed informed consent (or assent with guardian consent if under 18 years). Participants were excluded if they had a fever or had received any antimicrobial treatment within 24 h before the sample collection.

### 2.2 Study procedures

Participants attended a single visit where eligibility was confirmed, informed consent obtained. A structured questionnaire was used to gather demographic data (age, gender, residence), household characteristics (number of household members and highest parental education level), behavioral factors (school attendance, extracurricular activities, smoking exposure). Information on the meningococcal vaccination status of both the participant and their family members was also collected. Oropharygeal flocculated swabs were collected from every consented participant by trained healthcare workers and placed immediately in STGG (skim milk, tryptone, glucose, glycerine) medium. Samples were frozen at -80 °C within 4 h of collection and stored until processing. For meningococcal screening, samples were thawed and a 100 μL aliquot was inoculated onto GC VCAT (vancomycin, colistin, amphotericin, trimethoprim) selective agar plates (Oxoid, UK). Plates were incubated at 37 °C (±2 °C) in 5% CO₂ atmosphere and examined for potential meningococcal colonies after 16–24 h, with a second examination at 40–48 h. Suspected colonies were sub-cultured onto Columbia agar supplemented with 5% horse blood and incubated overnight (16–24 h). Colonies were subjected to oxidase testing (Pro-Lab Diagnostics, UK) and Gram staining. Oxidase-positive, Gram-negative diplococci underwent serogrouping using a dot-blot ELISA (32). Internal quality control procedures, including the use of control isolates, were applied throughout the process. Whole genome sequencing (WGS) was performed only on the three culture-confirmed oxidase-positive, Gram-negative diplococcal isolates using the Illumina MiSeq platform (33). No sequencing was performed directly on primary oropharyngeal swab specimens. Genomic DNA was extracted using the MAG-Bind Bacterial DNA kit (Omega Bio-tek, Inc., GA, United States). Paired end sequencing (2 × 300 bp) was performed using an Illumina MiSeq whereby library preparation was performed using the Illumina DNA Prep kit (Illumina, CA, United States). Draft genomes were assembled using Shovill (v1.0.4) with Spades (v3.14.1) with minimum contig length = 200 and minimum contig coverage = 10. Polymerase chain reaction (PCR)–based screening was not performed as part of the carriage detection workflow.

Assembled genomes were uploaded to (Neisseria spp.|PubMLST) for gene indexing.

### 2.3 Study endpoints

The primary endpoint of the study was to determine the proportion of participants with *N. meningitidis* as carriers among adolescents and young adults. Secondary endpoints included assessing the carriage prevalence across three age groups (15–16, 17–18, and 19–20 years), determining the serogroup distribution of *N. meningitidis* among carriers, and evaluating associations between carriage and demographic, geographical, behavioral, and vaccination factors.

### 2.4 Data analysis

The sample size was calculated based on an expected carriage rate of 5% with a precision of 2%, requiring 456 participants per age group. Data analysis included descriptive statistics, with categorical variables presented as frequencies and percentages and continuous variables as means, medians, and ranges. *N. meningitidis* carriage rates were analyzed using chi-square tests and logistic regression models to assess associations with demographic and behavioral variables.

### 2.5 Ethics statement

Ethical approval was obtained from the Standing Committee for Coordination of Health and Medical Research, Ministry of Health (Kuwait). Written informed consent was obtained from participants or legal guardians, with assent for those aged 15–17. The study was conducted in accordance with the principles of the Declaration of Helsinki, the International Ethical Guidelines for Biomedical Research Involving Human Subjects, and the International Conference on Harmonization (ICH) Guideline for Good Clinical Practice.

## 3 Results

**Figure 1:**
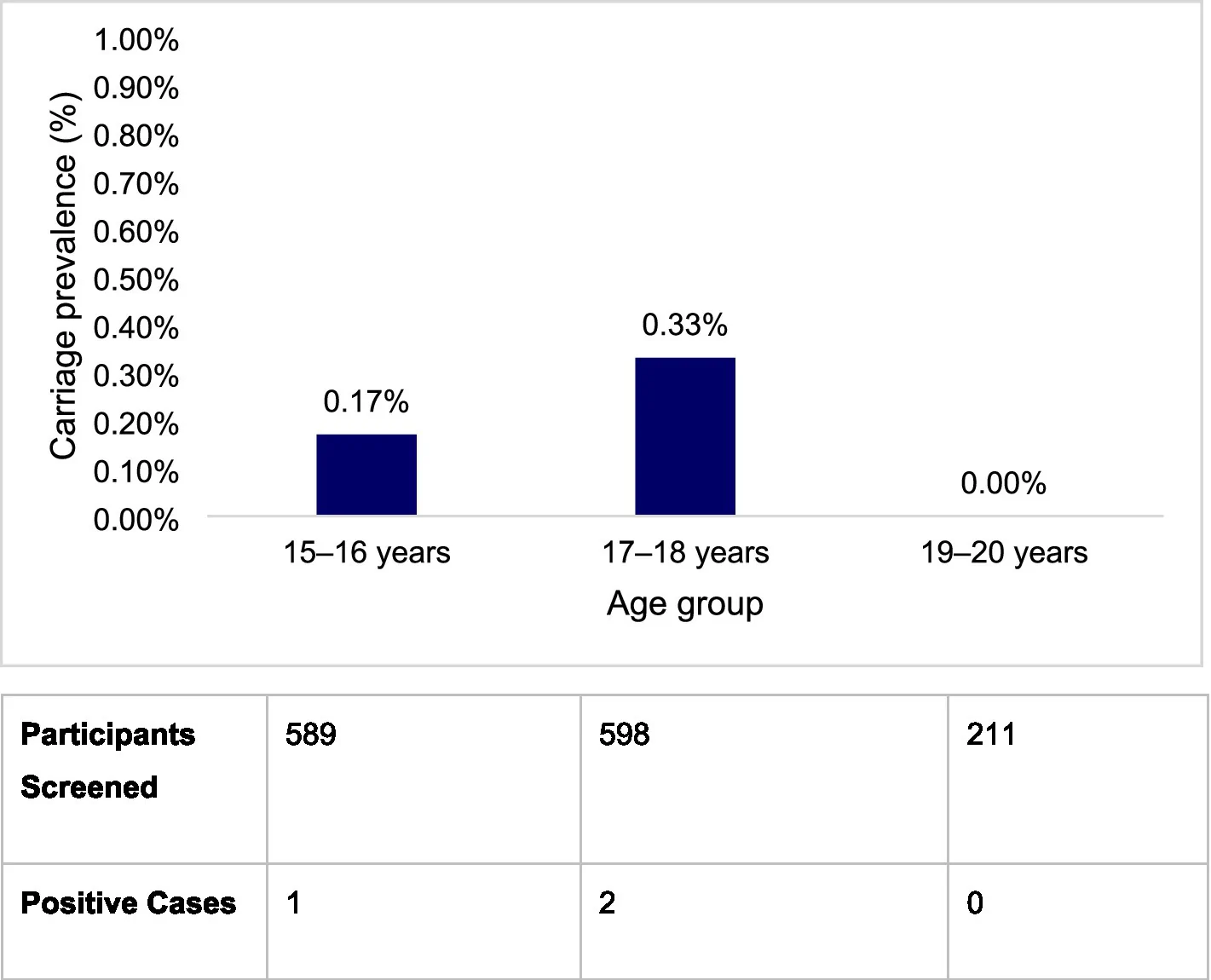
Distribution of *N. meningitidis* carriage by age group.

**Figure 2:**
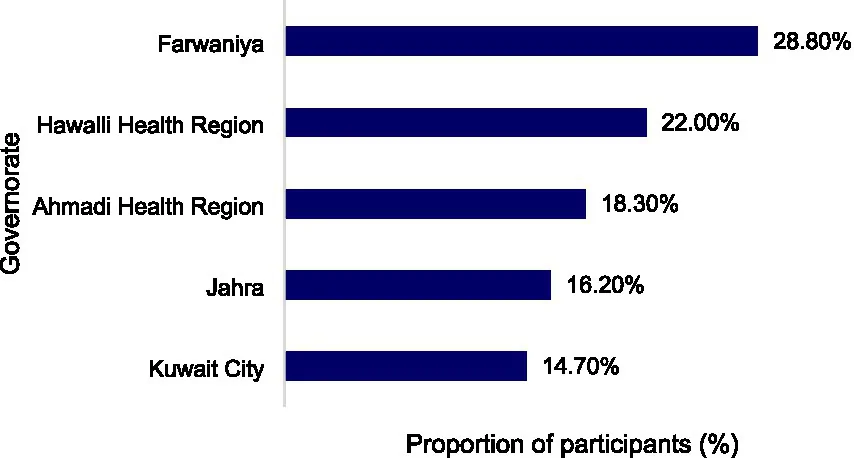
Geographic distribution of participants.

Among 1,398 participants, only three tested positive for *N. meningitidis*, yielding a carriage rate of 0.21% (3/1,398). By age group, one positive case was from 15–16 years (1/589) and two were from 17–18 years (2/598), and none among participants aged 19–20 years (0/211) (Figure 1). Participants were sampled from all six governorates, with the highest representation from Farwaniya Health Region (403/1,398, 28.8%) and Hawalli Health Region (308/1,398, 22.0%) (Figure 2). Most participants were adolescents aged 15–18 years (1,187/1,398, 85.0%), with 42% in the 15–16 years group and 43% in the 17–18 years group. The remaining 15% were young adults aged 19–20 years. Gender distribution showed a predominance of males (956/1,398, 68%), while females accounted for 31.6% (442/1,398). Regarding school attendance, 89.9% (1,257/1,398) of participants were actively attending school at the time of data collection, while 10% were not, which may reflect the study period overlapping with the summer break. In terms of extracurricular activities, 39.6% participated frequently (≥2 times per week), while 1.8% participated infrequently (≤4 times per month). (Table 1). Household cigarette smoking exposure was reported by 24.0% (336/1,398).

**Table 1:** Participant demographics.

Characteristic	*n* (%)
Age group	
15–16 years	589 (42%)
17–18 years	598 (43%)
19–20 years	211 (15%)
Gender	
Female	442 (32%)
Male	956 (68%)
School attendance	
Yes	1,257 (90%)
No	141 (10%)
Extra-curricular activities	
No	820 (58.6%)
Yes, frequent (≥2/week)	553 (39.6%)
Yes, infrequent (≤4/month)	25 (1.8%)
Household cigarette smoking	
Yes	336 (24%)
No	1,062 (76%)
Nationality	
Kuwaiti nationals	130 (9.4%)
Non-Kuwaiti residents	1,260 (90.6%)

**Figure 3:**
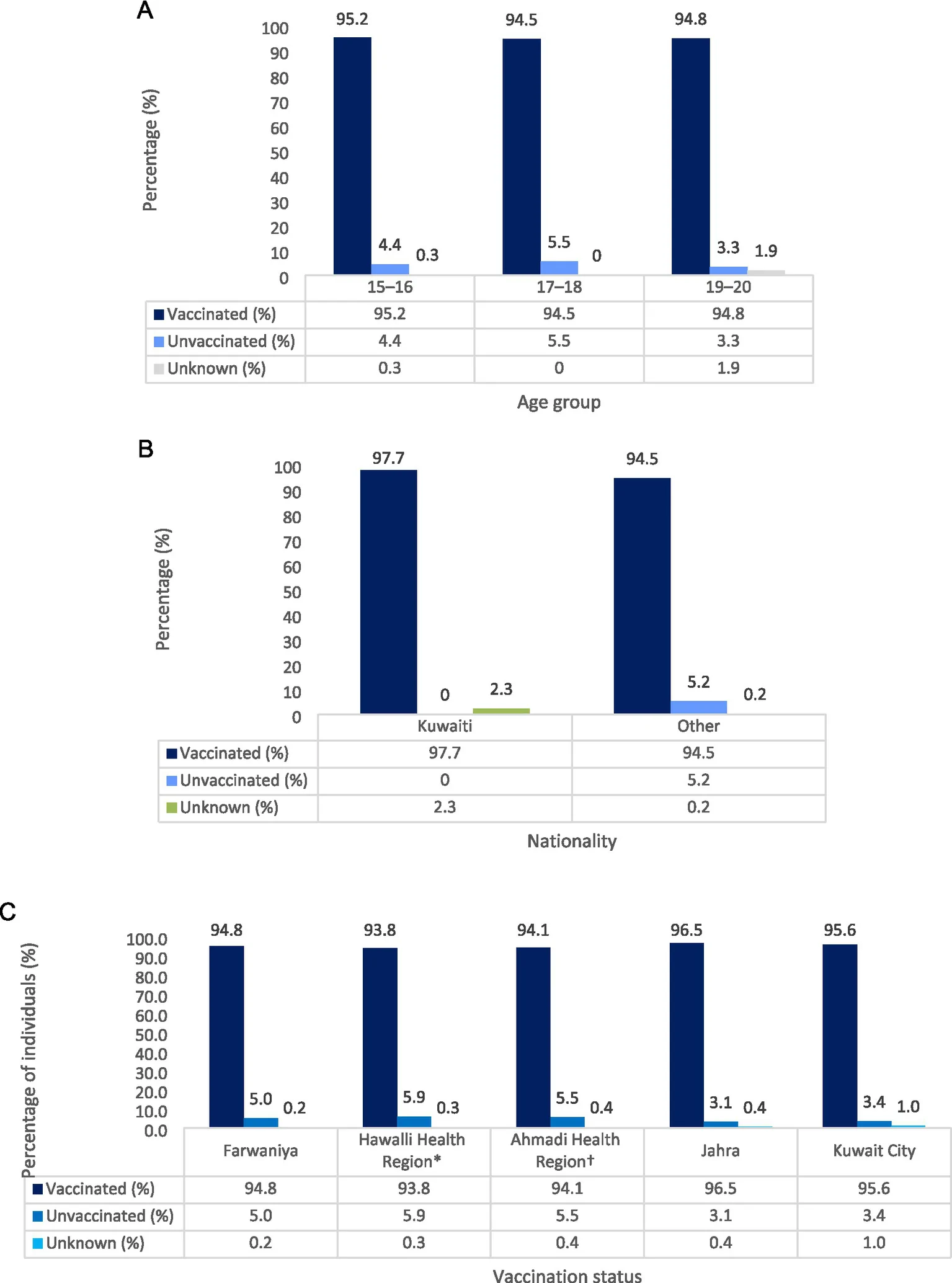
MenACWY vaccination coverage stratified by demographic characteristics. **(A)** Age group, **(B)** nationality, and **(C)** governorate of residence. Bars represent the proportion (%) of participants classified as vaccinated, unvaccinated, or with unknown vaccination status within each subgroup. **(A)** MenACWY vaccination coverage by age group. **(B)** MenACWY vaccination coverage by nationality. **(C)** MenACWY vaccination coverage by governorate of residence. * Hawalli Health Region = Hawalli + Salmiya. † Ahmadi Health Region = Ahmadi + Fahaheel.

All three isolates were non-groupable (NG), with one carrying the Capsule Null Locus (CNL) (Table 2). Self-reported MenACWY vaccination coverage was high, with 94.8% (1,326/1,398) of participants reporting being vaccinated, while 4.7% (66/1,398) were unvaccinated and 0.5% (6/1,398) were unsure of their vaccination status. When vaccination coverage was stratified by demographic characteristics, coverage remained consistently high across age groups, nationality, and governorates of residence. Vaccination coverage was similarly high among Kuwaiti and non-Kuwaiti participants and exceeded 90% across all major governorates, with only small proportions of participants reporting unvaccinated or unknown vaccination status (Figure 3).

**Table 2:** Characteristics of *N. meningitidis* carriage-positive participants.

Participant	Age group (years)	Gender	Species	Serogroup	Genogroup
1	17–18	M	*N. meningitidis*	NG	NG
2	15–16	F	*N. meningitidis*	NG	NG
3	17–18	M	*N. meningitidis*	NG	CNL

NG, Non-groupable; CNL, Capsule null locus.

## 4 Discussion

This study provides an estimate of *N. meningitidis* carriage among adolescents and young adults, revealing a low prevalence of 0.21%. It is notably lower than the 7–35% rates reported in unvaccinated adolescents (7, 34–36).

The high MenACWY vaccination coverage of 94.8% in our study population likely contributed to this low carriage rate, supporting the hypothesis that widespread immunization reduces *N. meningitidis* transmission. Studies in various countries have demonstrated that MenACWY vaccination significantly reduces *N. meningitidis* carriage, contributing to herd immunity and decreased transmission (4, 25, 37–40). For instance, the introduction of MenACWY vaccination in the United Kingdom has been linked to a substantial decline in carriage, leading to reduced disease incidence, particularly among adolescents (37). Similarly, data from Argentina and Chile support the claim that MenACWY vaccination leads to a decrease in *N. meningitidis* strains, such as MenW, suggesting a positive correlation between vaccination and reduced carriage (4). These results underscore the importance of high vaccine coverage in reducing the circulation of meningococcal serogroups. For example, in Carr et al. (37), carriage of genogroups C, W, and Y (combined) decreased from 2.03 to 0.71% [OR 0.34 (95% CI 0.27–0.44), *p* < 0.001]. However, carriage of genogroup B meningococci did not change [1.26% vs. 1.23% (95% CI 0.77–1.22), *p* = 0.80] and genogroup C remained rare (*n* = 7/10625 vs. 17/13438, *p* = 0.135). It is noteworthy that the *p*-values of this association demonstrate that the non-decrease in carriage of B and genogroup C was not statistically significant whereas the decrease C, W, and Y (combined) by *p* value was statistically significant. These findings are consistent with evidence suggesting an association between MenACWY vaccination and reduced carriage. However, some studies have found no significant correlation between MenACWY vaccination and reduced carriage (17, 41, 42). These discrepancies might be attributed to several factors such as varying vaccine coverage, differences in the population demographics (e.g., age, prior immunity), or methodological differences such as study design, sample size, or the diagnostic techniques used. MenB vaccination status was not collected, as MenB vaccines are not included in Kuwait’s national immunization program and are not routinely available. This could indicate naturally low carriage rates of MenB strains. The absence of MenB carriage in this study should therefore be interpreted cautiously, particularly given the very small number of carriage-positive isolates and the potential for under-detection of encapsulated strains using culture-based methods alone. Given that MenB has not been included in vaccination programs across many countries, further research is required to evaluate the role of herd immunity in reducing MenB prevalence (27).

Although our study did not include participants under 15 years of age, the observed low carriage rate may suggest indirect protection via herd immunity, likely influenced by routine MenACWY vaccination which was introduced in Kuwait in 1994 (27). The introduction of vaccination in early childhood has been shown to reduce carriage rates in adolescents, as evidenced by studies in the United Kingdom and other countries. These studies suggest that achieving herd protection, particularly through adolescent immunization programs, can substantially lower disease incidence and transmission (37, 43). A study demonstrated that previous vaccination, particularly during infancy and preschool years, was associated with a higher serum bactericidal antibody (SBA) response after a single booster dose at 11 years of age, compared to naïve adolescents. The highest SBA responses were observed in those vaccinated earlier, suggesting that early priming of the immune system leads to stronger immune responses in adolescence (44).

All detected isolates were non-groupable, including one carrying the capsule null locus, consistent with trends described in global reviews of meningococcal epidemiology (45). The absence of encapsulated serogroups may have implications for diagnostic methods and vaccine effectiveness assessments. Our study’s reliance on culture-based detection methods may have contributed to the identification of NG strains, which are often overlooked in conventional culture-based approaches (13). However, the limited number of positive samples restricted further characterization of circulating *N. meningitidis* strains. Given that NG strains are increasingly recognized as a challenge for vaccine strategies, this finding highlights the need for ongoing surveillance and the development of diagnostic techniques that can accurately identify NG strains.

Social and behavioral factors, such as smoking, overcrowding, and social interactions have been associated with increased *N. meningitidis* carriage. Smoking has been shown to increase susceptibility to respiratory infections, including *N. meningitidis* (9, 15, 46). In our study, 24% of participants reported exposure to household smoking, but the low overall carriage rate meant that it was not possible to establish a statistical association between smoking and carriage. Other demographic and social risk factors, including school attendance and extracurricular activities, could not be fully evaluated due to the low number of carriage-positive cases. Social behaviors are known to play a crucial role in *N. meningitidis* transmission (15, 46, 47). Notably, the lower carriage prevalence observed compared with Western settings may reflect differences in social mixing and living arrangements, as adolescents in Kuwait are more likely to reside within family households rather than shared dormitory or campus-based environments that are known to facilitate meningococcal transmission. Future studies with larger sample sizes and more comprehensive data are needed to explore these factors further.

## 5 Strengths and limitations

This study is the first population-based carriage study in Kuwait to evaluate the prevalence of meningococcal carriage at the population level using a random selection approach, ensuring a representative estimate of true prevalence and these are the strengths of the study.

Vaccination status was self-reported through participant questionnaires rather than verified through medical records, introducing substantial potential for recall misclassification and bias. While Kuwait has high vaccination coverage, undocumented vaccination history, particularly among non-nationals, may impact the accuracy of these data. The low number of meningococcal carriage-positive cases limited the ability to conduct meaningful subgroup analyses or establish significant associations with behavioral risk factors. Additionally, limited serogrouping data from broader IMD surveillance programs in Kuwait due to the low incidence restricts the comprehensive evaluation of vaccine impact and the potential need for additional serogroup specific immunization strategies. Although participants with recent antibiotic usage (within 24 h) were excluded, the potential effect of widespread antimicrobial overprescription as well as potential impact of antimicrobial resistance in the community on meningococcal carriage rates could not be assessed.

In Kuwait, IMD surveillance is based on mandatory reporting by healthcare facilities and laboratory confirmation, with parallel reporting streams designed to maximize case capture. While this system supports timely detection and monitoring of IMD, the very low national incidence limits the yield of serogroup-specific epidemiological data, which restricts contextual interpretation of carriage findings.

An additional limitation is that the questionnaire did not capture the specific meningococcal vaccine received. Vaccination status was self-reported and not verified against medical records, which may have introduced recall misclassification. MenB vaccines are not registered and are not available in either the public or private healthcare sectors in Kuwait; therefore, the number of participants likely to have received a MenB vaccine would be expected to be extremely small. As a result, MenB vaccination status was not systematically collected, and its potential impact on carriage could not be assessed in this study.

## 6 Conclusion

In this population-based sample of 1,398 adolescents and young adults in Kuwait, *N. meningitidis* carriage prevalence was 0.21%. All detected isolates were non-groupable, including one carrying the capsule null locus. Self-reported MenACWY vaccination coverage was 94.8%, and no statistically robust associations with demographic or behavioral factors could be identified due to the small number of carriage-positive participants. Future studies should include registry-verified vaccination status, meningococcus-specific detection workflows, and linkage with national IMD surveillance data.

## Data Availability

The datasets presented in this study can be found in online repositories. The names of the repository/repositories and accession number(s) can be found at: https://pubmlst.org/organisms/neisseria-spp, 154700.
