# Correction: Meningococcal carriage in adolescents and young adults in Kuwait

**DOI:** 10.3389/fpubh.2026.1966089

**Published:** 2026-08-25

**Authors:** Mohammad Alghounaim, Hamad Bastaki, Mohamed Egaila, Jennifer Onwumeh-Okwundu, Mohammed Abdel Aziz, Jean Joury, Lidia Sierra, Florence Lefebvre D'Hellencourt

**Affiliations:** 1 Department of Pediatrics, Amiri Hospital, Kuwait City, Kuwait; 2 Department of Public Health, Ministry of Health, Kuwait City, Kuwait; 3 Pfizer Gulf FZ LLC, Dubai, United Arab Emirates; 4 RWE Platform Pfizer Inc., Collegeville, PA, United States; 5 Pfizer Inc., New York, NY, United States

**Keywords:** Kuwait, meningococcal carriage, Neisseria meningitidis, public health, vaccination

In the **Acknowledgments**, the following text was inadvertently omitted from the original publication:

Screening of oropharyngeal swabs, meningococcal serogrouping, and genome sequence analysis were performed at the UKHSA Meningococcal Reference Unit. Illumina sequencing was performed at the UKHSA Pathogen Genomics Sequencing Laboratory, Manchester.

Revised **Acknowledgments:** “The authors would like to acknowledge Hiral Joshi, Pharm D., and Axa Jacob, Pharm D. for their medical writing and editorial support for developing this manuscript. They also acknowledge Hammam Harridy, Graciela Morales, and Elvera Campbell for the financial support and Agatha Strozyk for acting as research collaboration study manager on this project. Screening of oropharyngeal swabs, meningococcal serogrouping, and genome sequence analysis were performed at the UKHSA Meningococcal Reference Unit. Illumina sequencing was performed at the UKHSA Pathogen Genomics Sequencing Laboratory, Manchester.”

The original version of this article has been updated.

